# Trade and investment liberalization, food systems change and highly processed food consumption: a natural experiment contrasting the soft-drink markets of Peru and Bolivia

**DOI:** 10.1186/s12992-016-0161-0

**Published:** 2016-06-02

**Authors:** Phillip Baker, Sharon Friel, Ashley Schram, Ron Labonte

**Affiliations:** School of Regulation and Global Governance (RegNet), The Australian National University, Canberra, ACT 2601 Australia; School of Epidemiology, Public Health and Preventive Medicine, University of Ottawa, Ottawa, ON K1H 8M5 Canada

**Keywords:** Trade and investment, Latin America, Soft-drinks, Natural experiment, Non-communicable diseases

## Abstract

**Background:**

Free trade agreements (FTAs) can affect food environments and non-communicable disease risks through altering the availability of highly-processed foods. Few studies have quantified such effects. Using a natural experiment this paper quantifies changes in Peru’s soft-drink market before/after entry into the US-Peru FTA, compared with Bolivia, a county with no such agreement.

**Methods:**

Difference-in-difference models were used to test for between country differences in the rate of per capita foreign direct investment (FDI) inflows, soft-drink imports, the volumes of various soft-drinks sold, and the volumes of sugar from soft-drinks before/after FTA ratification (2006) and enforcement (2009).

**Results:**

In Peru average per capita FDI-inflows rose from US$103.11 in the pre-ratification period to US$269.79 post-ratification, with little change in Bolivia. This corresponded with a 122 % increase in Peruvian soft-drink production. There was a significant between-country difference in FDI-inflows pre-/post-ratification (DID:1.07, 95 % CI:0.19–1.96; *p* = 0.01). Despite little difference in total per capita soft-drink sales volumes there was a significant between-country difference in per capita sugar from soft-drinks pre-/post enforcement (DID:-0.99, 95 % CI: −1.91–0.06; *p* = 0.03) with stagnated growth in Peru and continued growth in Bolivia. This resulted from stagnated sugar sweetened carbonates growth and increased bottled water, juice and sports & energy drinks growth in Peru, with continued carbonates growth in Bolivia. There was a significant between-country difference in per capita carbonates (DID: −1.44, 95 % CI: −2.52–0.36, *p* = 0.01) and bottled water (DID:0.63; 95 % CI: −0.01–1.26; *p* = 0.04) sales volumes.

**Conclusions:**

The FTA may have resulted in increased FDI-inflows and soft-drink production and also contributed to the diversification of soft drinks produced and sold in Peru with some positive (stagnated carbonates and increased bottled water) and some negative (increased juice and sports & energy drinks) implications for nutrition. These changes were not evident in Bolivia. These results should be interpreted cautiously given the study design limitations.

## Background

This paper aims to inform understanding of the impacts of trade and investment liberalization events on population-level nutrition by quantifying the effects of the United States-Peru Trade Promotion Agreement (US-Peru FTA) on Peru’s soft-drink market and consumption, in contrast to Bolivia, a country with no such agreement.

Since the 1980’s the proliferation of free trade agreements (FTAs) has globalized markets in highly-processed foods. Because such foods (e.g. biscuits, confectionary, savoury snacks and sugar sweetened beverages), tend to be high in glycaemic load, fat and salt and because their consumption is rapidly increasing in low- and middle-income countries (LMICs), they are implicated in the rising burden of diet-related non-communicable diseases (NCDs) globally [1, 2].

There are several reasons why the globalization of markets in sugar-sweetened beverages (SSB) in particular is important to the investigation of trade agreements and health. There is strong empirical evidence implicating SSB consumption as a risk factor for obesity, type-2 diabetes and CVD [3, 4]. Transnational beverage companies (TBCs) are, in terms of sales and market capitalization, among the largest and most economically powerful group of transnational food and beverage companies [5, 6]. The sector is highly concentrated at the global level with two US firms, Coca-Cola and PepsiCo, together controlling 35.7 and 71.7 % (by value) of the soft-drink and carbonated soft-drink markets respectively in 2014 [7]. Through their considerable market power, including a combined $7.27 billion global advertising expenditure in 2013 [8], these firms can shape global and local food systems in ways that alter the availability, affordability and desirability of soft-drinks and thereby shape population level consumption patterns [9–12].

Evidence demonstrates that the evolving global trade regime creates favourable market conditions that allow TBCs to more easily transfer investments, technologies, production capacity, raw materials and final products from high-income to LMICs [10, 13–16]. Recently there has been concern that the emerging form of regional FTAs moves the scope beyond reducing tariffs on imports to increased protections for foreign investors and a deepening reach ‘behind-the-border’ into regulatory controls on governments domestic policy-making capacities [17, 18].

The Trans Pacific Partnership (TPP) agreement between 12 ‘Pacific-Rim’ nations, is potentially the most significant trade policy of the 21st century, representing a population of 792 million consumers and 40 % of global gross domestic product [19]. Negotiations for the TPP were completed in October 2015 and a final draft text of the full agreement released only in early November 2015. The TPP is likely to have serious implications for population-level nutrition and health [20]. Recent qualitative analyses of previously leaked chapters of the negotiating text revealed provisions on foreign investment liberalization (including stronger investor protections) and intellectual property rights that extend beyond those of existing multilateral agreements. Such provisions have the potential to facilitate greater TBC market access and promote the availability and consumption of highly-processed foods, while simultaneously restricting the capacities of governments to regulate markets for these foods to protect public health [18, 21].

However to date there has been little quantitative evidence as to the likely nutritional effects of FTAs such as the TPP. Stuckler et al. found that per capita soft-drink consumption was 63.4 % higher in countries with a United States (US) FTA than in countries with no such agreement. The same study reported a large increase in soft-drink consumption in Mexico following the North American Free Trade Agreement (NAFTA) in 1994, which by 2010 had reached 300 litres per capita per year—the highest volume globally [10]. Others also observed similar increases in unprocessed and processed food imports and foreign direct investment (FDI) by US firms into Mexico following NAFTA, including soft-drinks [22]. Large-scale changes in unprocessed and processed food imports and consumption were also observed in Central American countries following the US-Central America Free Trade Agreement (CAFTA) in 2004 [23]. Another study found significant associations between liberalization measures and diet-related health outcomes including cardiovascular diseases (CVD) and obesity in Sub-Saharan Africa [24]. In a previous analysis we found that the removal of restrictions on FDI by Vietnam, following its accession to the WTO in 2007, led to significant growth of that countries sugar-sweetened carbonated beverages market, in contrast to the Philippines a matched control country that had acceded in 1995 [25]. These findings are consistent with evidence of a positive association between FDI and the prevalence of diet-related NCDs in middle-income countries [10].

The motivation for this analysis was to elucidate the effects of a previously ratified FTA with the aim of better understanding the potential implications of new FTAs, including the TPP, for population nutrition.. Additionally, methodologies for quantifying the effects of FTAs on nutrition are underdeveloped and we set out to determine whether a natural experiment design, commonly used to assess the effects of policies and other large-scale interventions, might be appropriate [26]. On these grounds we adopted a natural experiment design to quantify the effects of the US-Peru FTA, ratified in 2006 and enforced in 2009, in contrast to Bolivia a suitably-matched country having no such agreement, on Peru’s soft drink market. The US-Peru FTA, which is similar to the preceding NAFTA and CAFTA agreements, resulted in the preferential elimination of tariffs on soft-drink imports (previously 25 %), as well as stronger protections for US investors, and strengthened intellectual property rights [27]. Obesity is a pressing public health concern in Peru and Bolivia where rates of adult obesity are high, 15.7 and 17.9 % respectively [28, 29]. By soft drinks we refer to the sugar-sweetened/high-sugar categories carbonates, juice and sports & energy drinks, and the unsweetened category bottled water.

In this analysis we test the following three hypotheses;

- Hypothesis One: Reduced barriers to investment resulted in a significant change in FDI-inflows into Peru, with no comparative change in Bolivia, and a corresponding change in soft-drink production in Peru.
- Hypothesis Two: Reduced barriers to trade resulted in a significant change in soft-drink imports into Peru, with no comparative change in Bolivia.
- Hypothesis Three: Changes in FDI-inflows, production and imports resulted in a significant change in soft-drink sales volumes in Peru, corresponding with a change in sugar from soft-drinks, with no comparative respective changes in Bolivia.

## Methods

### Study design and case selection

A natural experiment study design was adopted [26, 30] since the intervention (ratification of a US-FTA) is not amenable to experimental manipulation, and it was possible to compare an intervention country (one that had ratified a US-FTA) and a control country (one that had not) [26]. The control country was matched to the intervention country against economic, demographic and trade indicators reported in the literature to effect soft-drink consumption [3, 10], including population and income growth (Table 1). We also wanted to include a TPP country in the analysis to demonstrate what has previously happened in the country as a consequence of trade liberalization, thereby providing a richer understanding of the context in which the TPP will operate. Against these criteria Peru was selected as the intervention country and Bolivia as the control country.

**Table 1:** Change in demographic, economic and trade indicators for Peru and Bolivia, 1993–2013

	Peru				Bolivia			
	1993	2003	2013	∆ 1993–2013 %	1993	2003	2013	∆ 1993–2013 %
Population (millions)	23.08	27.07	30.38	31.63	7.3	9.0	10.2	39.73
Urban population (% of total)	70	74	78	11.43	58	63	68	17.24
GDP per capita, PPP	5,329	6,883	11,396	113.85	3,887	4,367	5,934	52.66
FDI intensity (% of GDP)	2.2	2.3	4.6	109.09	2.2	2.4	5.7	159.09
Trade intensity (% of GDP)	29	37	48	65.52	47	52	81	72.34

*Footnotes:* GDP per capita expressed as purchasing power parity (PPP) in constant 2011 international $ for comparability; Data from World Bank World Development Indicators; ∆ = change

The US-Peru FTA was ratified in 2006 and enforced in 2009. We predicted that due to enhanced US investor confidence in Peru (a so-called ‘market signal’) following ratification, trade and investment flows and changes in soft-drink markets may have begun prior to enforcement. Additionally, the agreement may not have had immediate effects given the potential time-lag between increased capital investments in plants and machinery and production outputs resulting from those investments. To ascertain the effects of timing, therefore, we used two intervention time points in our statistical tests corresponding to the ratification year 2006 and the enforcement year 2009 respectively. Exogenous historical variables may have generated some heterogeneity in the data [30]. To contextualise the analysis we therefore prepared a description of significant trade and investment liberalization events and market developments in the respective countries, as given in Table 2.

**Table 2:** Trade and investment liberalization events and market developments in Peru and Bolivia

Both countries have progressed through successive stages of trade liberalization namely protectionism, unilateral liberalization, accession to the multilateral system (WTO) and more recently bilateral and regional free trade agreements.	
Peru	
*Unilateral liberalization:* Prior to the 1990s high levels of protectionism existed with high tariffs and import bans, with a 66 % tariff average in 1989, and strict controls on foreign investment. Unilateral liberalization began in 1990 through extensive economic reforms. This included tariff reductions (maintaining a 25 % tariff on soft-drinks), the elimination of most import prohibitions, strengthened customs procedures, the privatization of state-owned food enterprises, and the establishment of independent institutions to regulate market competition, intellectual property rights, and foreign investment. In 1993 foreign capital flows and currency exchanges were liberalized. By 1998 the average tariff rate was 13.5 % with no import prohibitions in place.	
*Multilateral liberalization:* Already a member of the General Agreement on Tariffs and Trade (GATT) system, on January 1st 1995 Peru acceded to the WTO. Under Peru’s General Agreements on Trade in Services (GATS) commitments, sectors relevant to processed foods including advertising services, wholesale trade services of beverages, and retailing services of beverages were fully liberalized with the exception of commercial advertising material produced outside of Peru.	
*Regional and bilateral liberalization:* Since 2004 Peru has entered into multiple PTAs including intra-regional (e.g. MERCOSUR), extra-regional (e.g. European Union) and bilateral (e.g. US) agreements. The US-Peru FTA was the second of such agreements (the first being MERCOSUR), but is likely to have had the most significant implications for Peru’s soft-drink market given that the two largest transnational soft-drink companies (Coca-Cola Company and Pepsico) are US firms. It is also similar to CAFTA and NAFTA (i.e. containing similar provisions) and these agreements had demonstrated impacts on processed food consumption in previous analyses [27, 40, 23]. The US-Peru FTA eliminated a 25 % tariff on soft-drinks for US firms, while the average MFN tariff rate (i.e. for other WTO trade partners) was 6 % in 2013.	
*General market description and developments:* Prior to 1999 the US-firm Coca-Cola Company (CCC) and the Peruvian-firm Corp JR Lindley were the two major competitors in the Peruvian soft-drink market. The two leading brands were Coca-Cola and Inca Kola. Today the soft-drink market is dominated by two firms. The first was formed in 1999 through a US$200 million merger between the CCC and Corp JR Lindley (CCC-CRL), and had a total market share of 49.8 % in 2013. The second, the Peruvian firm Aje Group, had a market share of 29.7 % the same year. A third Pepsi-Cola Panamericana Peru SRL, a subsidiary of the US-firm Pepsico, had a market share of 9.2 %. CCC-CRL has made a number of significant investments in domestic production and bottling facilities, including acquisition of the bottler Embotelladora Latinonamericana in 2004, and further investments in ‘mega’ production plants and distribution facilities. In 2014, the CCC announced a US$1 billion, 5-year, investment in its Peruvian operations. Prior to 2006 the Peruvian soft-drink market was undiversified with firms competing for market share of the carbonates market. Since then, both CCC-CRL and Aje group have diversified their product mix, with significant increases in sales of bottled water, juice, and sports & energy drinks.	
Bolivia	
*Unilateral liberalization:* Following a long period of economic instability, in 1985 a newly-elected Estenssoro Government implemented its *New Economic Policy* that transformed Bolivia from a relatively inward-orientated state-capitalist economy to a more liberalized and privatised one. This included the elimination of quantitative restrictions on imports and the adoption of a uniform 10 % tariff rate. In 1993 under the ‘capitalization’ agenda of the Lozada Government, foreign investment restrictions were relaxed and many state-owned enterprises underwent partial privatization.	
*Multilateral liberalization*: As a member of the GATT system in September 1995 Bolivia acceded to the WTO. Subsequently in December 1996 Bolivian legislation was implemented to establish free importation with no prior licensing, import quotas or other non-tariff measures. Bolivia’s average applied MFN tariff rate fell from 9.7 % in 1999 to 8.2 % in 2005, with few non-tariff barriers. Under the GATS agreement Bolivia made commitments in five of the 12 sectors covered, although sectors most relevant to processed foods were excluded.	
*Regional and bilateral liberalization:* Bolivia is a member of the Andean Community (a custom’s union with Colombia, Ecuador and Peru). It has entered into preferential trade agreements with Mexico and the MERCOSUR group of countries. As a member of the Bolivarian Alternative for the Americas (ALBA) Bolivia aligns politically with other socialist governments in the region (Cuba, Ecuador and Venezuala). This group partly aims to create alternative regional trade initiatives. In 2008, Bolivia withdrew from trade talks with the European Union and later from talks with the United States and Canada.	
*General market description and developments:* Through franchising arrangements the US-firm Coca-Cola Company (CCC) has dominated Bolivia’s soft drink market, with a commercial presence since 1943. In 1995 the franchise Vascal and various bottling operators were acquired by Embotelladoras Bolivianas Unidas SA (EMBOL), a CCC subsidiary, using capital provided by Coca-Cola Embonor (Chile). In 1997, Coca-Cola Embonor acquired an additional 18.7 % of EMBOL to control 99.9 % of the subsidiary in Bolivia. EMBOL owns seven factories with an annual production capacity of 155 million unit cases (approx. 880 million litres) for its 12 brands. Through its distribution network the company supplies 75,000 retailers. This infrastructure makes EMBOL, and thus the CCC, the largest manufacturer and distributor of soft drinks in the country with an overall soft drinks market share of 58.3 % in 2013. Cerveceria Boliviana Nacional is the national franchise manufacturer and distributor of Pepsi products, and the second leading market player with a market share of 17.2 % in 2013.	

### Conceptual framework

**Fig. 1:**
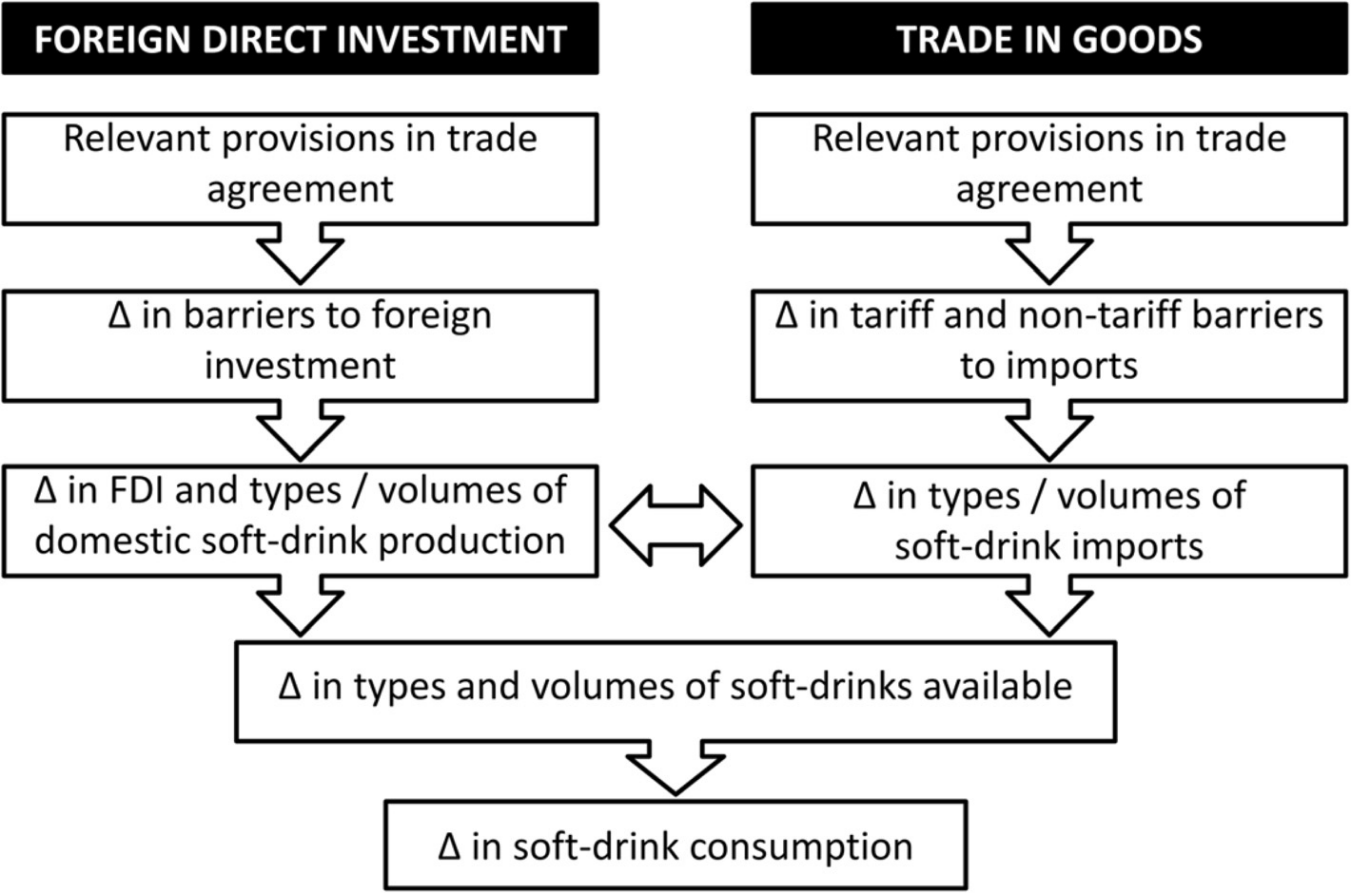
The ‘foreign direct investment’ and ‘trade in goods’ conceptual pathways used to structure the data analysis. *Footnotes:* ∆ = change; Adapted from [27]

The analysis was guided by a recent conceptual framework developed as part of the INFORMAS trade and nutrition monitoring module [31]. Specifically, the ‘foreign direct investment’ and ‘trade in goods’ pathways were used, as described in Fig. 1. Based on these pathways we assigned ratification and enforcement of the agreement as the independent variable, and FDI-inflows, soft-drink import volumes, soft-drink sales volumes and sugar from soft drinks volumes as the dependent variables.

### Variables, measures and data sources

A description of variables, measures and data sources used in the analysis is given in Table 3. All economic, FDI, production and trade data were adjusted as per capita, using total population estimates from the World Bank World Development Indicators database [32]. Per capita soft-drink sales volumes were extracted from Euromonitor International Passport Global Market Information database and used as a proxy measure of consumption for the various product categories given in Table 3 [7]. Market growth data were also extracted as % year-on-year sales growth for the same categories. A ‘Sugar from Soft Drinks’ variable was included as a proxy measure of sugar consumption from soft-drinks. We have previously described the method for calculating this using Euromonitor ingredients data linked to the soft-drink categories [33]. Euromonitor collects sales data from a number of sources including trade associations, industry bodies, business press, company financial reports, company filings and official government statistics. Consumption volume estimates are validated by people working within the food industry [7].

**Table 3:** Description of variables and measures included in the analysis, with data sources

Variable	Measure	Years	Definition	Source
Per capita gross domestic product	Purchasing power parity, constant 2011 international $; % year-on-year growth	1990–2013	Gross domestic product converted to international dollars using purchasing power parity rate for comparability	[32]
Per capita foreign direct investment in-flows	US$ millions, fixed 2013 prices and exchange rates; % year-on-year growth	1990–2013	Value of inward investments involving long-term relationships and reflecting lasting interests in and control by resident (parent) entities in one economy of (affiliate) enterprises resident in a different economy	[39]
Per capita soft-drink production	US$ millions, fixed 2013 prices and exchange rates	2000–2013	Value of manufactured non-alcoholic beverages, except non-alcoholic beer and wine, and of the production of natural mineral waters	[7]
Per capita soft-drink imports	Litres; % year-on-year growth	1999–2012	Import volume of mineral waters and aerated waters containing added sugar or other sweetening matter or flavoured	[41]
Per capita soft-drink sales	Sales in litres; % year-on-year growth	1999–2013	Sales volume of the aggregation of all beverage categories	[7]
Per capita carbonates sales	Sales in litres; % year-on-year growth	1999–2013	Sales volume of cola and non-cola carbonates, whether regular or low calorie, containing dissolved carbon dioxide, regular & low calorie	[7]
Per capita bottled water sales	Sales in litres; % year-on-year growth	1999–2013	Sales volume of still bottled water, carbonated bottled water, flavoured bottled water and functional bottled water	[7]
Per capita juice sales	Sales in litres; % year-on-year growth	1999–2013	Sales volume of 100 % juice, nectars (25–99 % juice content), juice drinks (up to 24 % juice content), fruit-flavoured drinks, and cereal/pulse-based drinks	[7]
Per capita sports & energy drinks sales	Sales in litres; % year-on-year growth	1999–2013	Sales volume of sports and energy drinks	[7]
Per capita sugar from soft-drink volume	Volume in kilograms; % year-on-year growth	2000–2013	Aggregated volume of sucrose, glucose, fructose (including high fructose corn syrup) used in the manufacture of soft-drinks	[7]

### Statistical analysis

To test the hypotheses we ran a series of difference-in-difference (DID) models [30], using STATA v13. This utilized the cross-sectional time-series data described earlier to assess the statistical significance of the difference between the intervention country (Peru) and control country (Bolivia) within time-period differences as defined by the two pre- and post-intervention time points.

For H1 we ran a DID model comparing between-country changes in FDI-inflows per capita (1). Because soft-drink production data was unavailable for Bolivia we were unable to run a DID model comparing changes in production and instead provide the descriptive statistic for Peru only. For H2 we ran a DID model comparing between-country changes in the rate of beverage imports from all trading partners. For H3 we ran a DID model comparing between-country changes in the rate of soft-drink sales volumes (3a) and sugar from soft-drinks volumes respectively (3b). In addition to the DID models, time-series line graphs were included to assist in visualizing time trends. Model specifications are given below, where T1 represents estimates in the pre-intervention period; T2 represents estimates in the post-intervention period; FDI represents foreign direct investment in-flows per capita; IMP represents rate of soft-drink import volumes per capita; SSV represents rate of soft drink sales volumes per capita; and SUG represents per rate of sugar from soft drinks volume per capita, respectively:

- ΔΔFDI = (ΔFDI_Peru_[FDI_T2_–FDI_T1_] – ΔFDI_Bolivia_[FDI_T2_–FDI_T1_])
- ΔΔIMP = (ΔIMP_Peru_[IMP_T2_–IMP_T1_] – ΔIMP_Bolivia_[IMP_T2_–IMP_T1_])
- ΔΔSSV = (ΔSSV_Peru_[SSV_T2_ − SSV_T1_] – ΔSSV_Bolivia_[SSV_T2_–SSV_T1_])
- ΔΔSUG = (ΔSUG_Peru_[SUG_T2_ − SUG_T1_] – ΔSUG_Bolivia_[SUG_T2_–SUG_T1_])

We also included income (measured as GDP per capita) as a co-variate, as this was identified as strongly associated with soft-drink consumption in previous analyses [3, 10]. Population growth was implicitly controlled for by converting all data to per capita. The DID model is also prone to error arising from auto-correlation of the time-series data and from heterogeneity in the dependent variable arising from the effects of exogenous variables. To minimise this we controlled for auto-correlation using time in years as a covariate. Log_e_ transformations were performed on heteroskedastic variables (determined by comparative distribution plots and tests for heteroskedascity). Robust standard errors were calculated for all model outputs, from which we calculated ±95 % confidence intervals.

## Results

### Comparing changes in FDI-inflows and soft-drink production

**Fig. 2:**
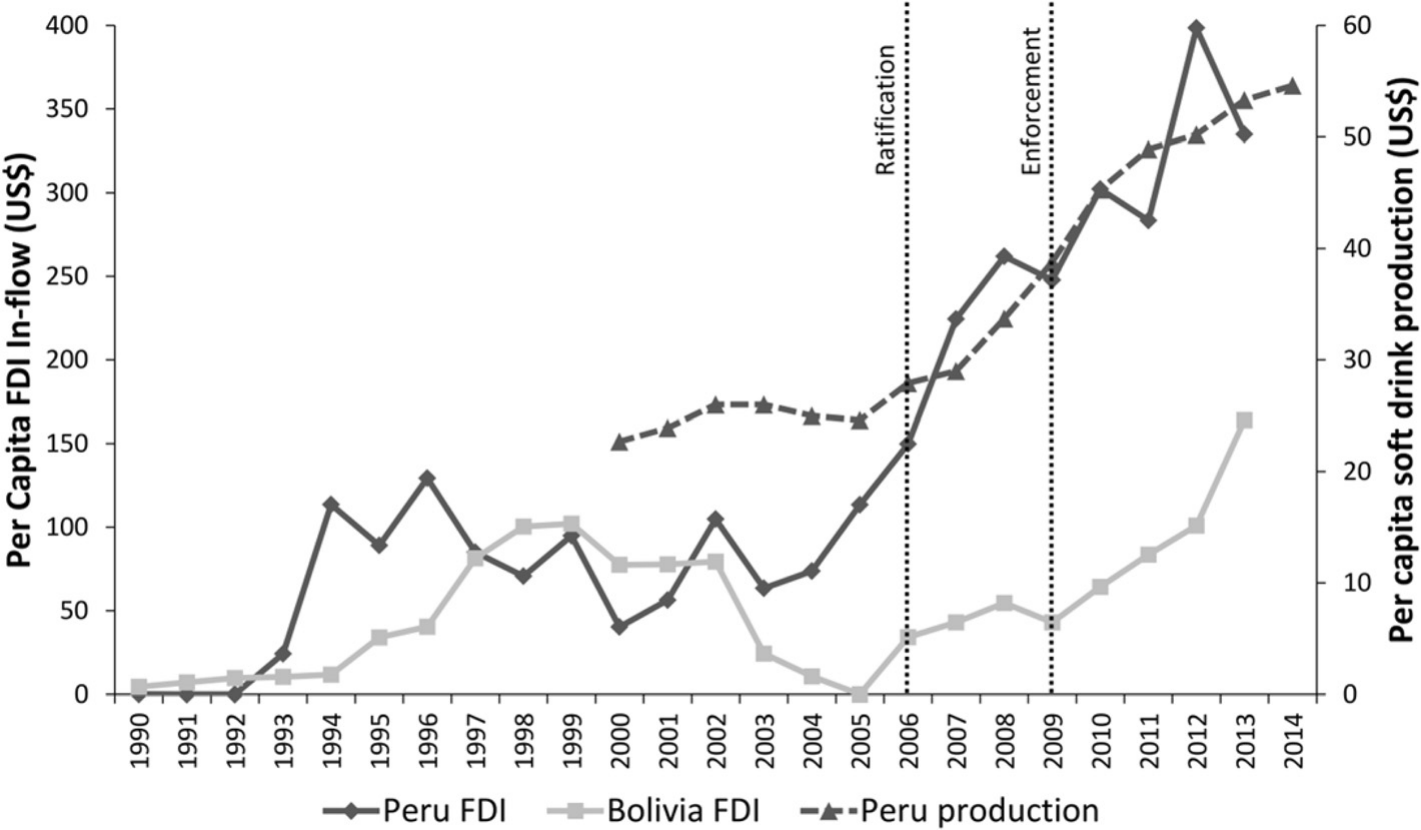
Trends in per capita FDI-inflows into Peru and Bolivia, and per capita soft-drink production in Peru, before and after ratification (2006) and enforcement (2009) of the US-Peru FTA. *Footnotes:* FDI in-flow data in US$ (fixed 2013 exchange rates and prices) from [36, 39]; soft-drink production data in US$ (fixed 2013 exchange rates and prices) from [19]

The first hypothesis was that reduced barriers to investment would result in a significant change in FDI-inflows into Peru following the FTA and a corresponding change in soft-drink production. Figure 2 demonstrates trends in FDI-inflows per capita in Peru and Bolivia. In Peru average per capita FDI-inflows rose from US$103.11 (95 % CI: 67.24–139.98) in the pre-ratification period to US$269.79 (95 % CI: 225.00–314.58) in the post-ratification period. In Bolivia average FDI-inflows per capita declined 11.45 % from US$93.39 (CI: 50.23–136.55) and US$82.69 (95 % CI: 67.75–97.63) respectively. The DID model, with an intervention time-point of 2006, revealed a significant between-country difference in log_e_ FDI-inflows per capita (1.07, 95 % CI: 0.19–1.96; *p* = 0.013). This was robust to adjustments for GDP and underlying time-trends (Table 4). The observed change in FDI-inflows corresponded to a 122 % increase in soft-drink production in Peru from US$24.60 per capita in 2005 to US$54.60 per capita in 2014 (Fig. 2).

**Table 4:** Between-country difference-in-difference estimates for rate of change in log_e_ FDI in-flows per capita before and after ratification (2006) and before and after enforcement (2009) of the US-Peru FTA

Year	Estimate	Unadjusted	Adjusted for GDP	Adjusted for GDP & time trends	N
2006	Diff-in-diff	1.07** (0.43)	1.03** (0.47)	1.03** (0.50)	28
	R^2^	0.63 (*p* = 0.013)	0.64 (*p* = 0.028)	0.65 (*p* = 0.041)	
2009	Diff-in-diff	0.68* (0.37)	0.67* (0.40)	0.68 (0.43)	28
	R^2^	0.53 (*p* = 0.064)	0.56 (*p* = 0.091)	0.60	

*Notes:* *p < 0.1; **p < 0.05; robust standard-errors in parentheses

### Comparing changes in soft-drink imports

**Fig. 3:**
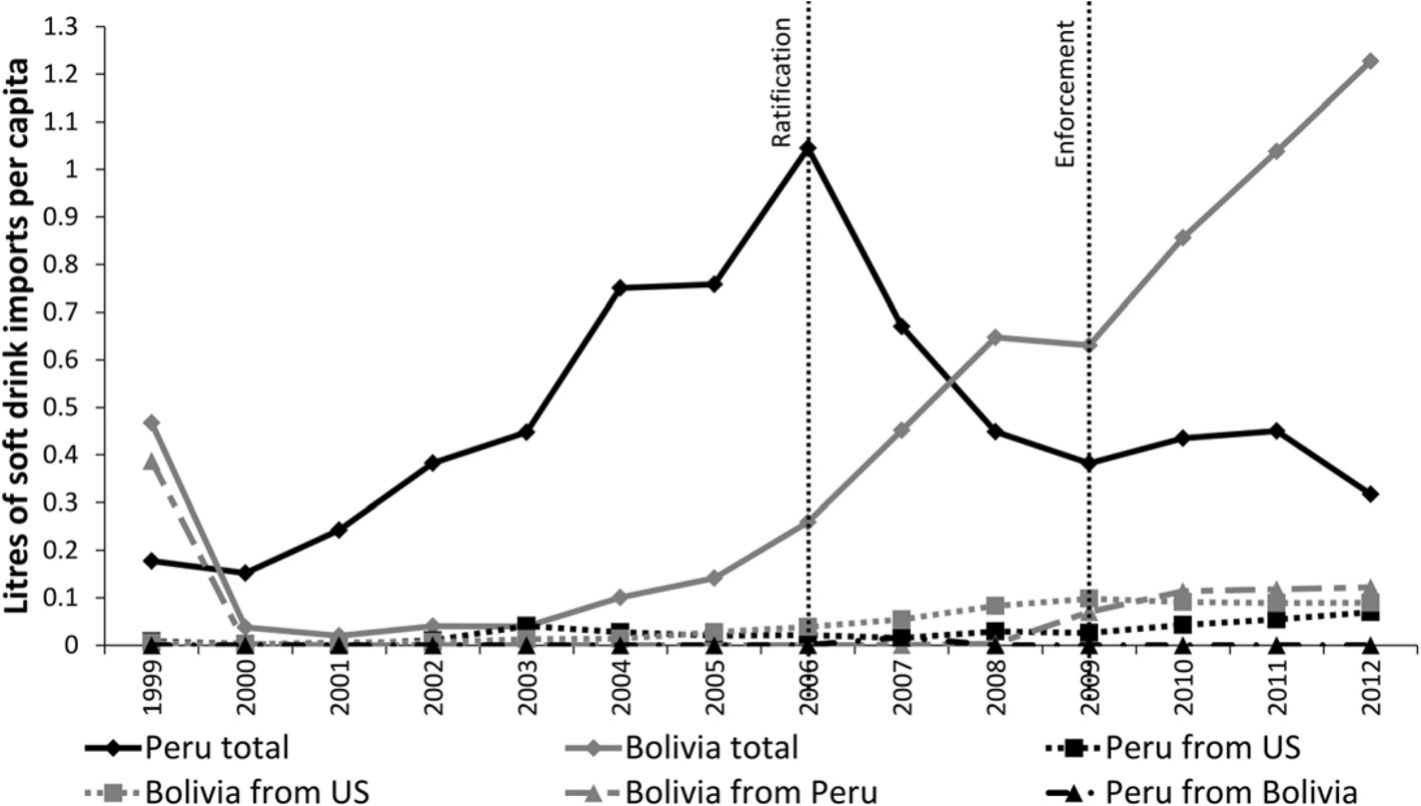
Total soft-drink imports (from all trading partners), before and after ratification (2006) and enforcement (2009) of the US-Peru FTA. *Footnotes:* Data from [37]

The second hypothesis was that reduced barriers to trade would result in a significant change in soft-drink imports into Peru. Figure 3 demonstrates trends in per capita soft-drink imports. In Peru average per capita total soft-drink imports increased from 0.42 L (95 % CI: 0.21–0.63) in the pre-ratification period to 0.54 L (95 % CI: 0.35–0.73) post-ratification, although between 2006 and 2009 imports declined. The same figures for Bolivia were 0.12 L (95 % CI: 0.00–0.24) and 0.73 L (95 % CI: 0.46–1.00) respectively. The DID model, with an intervention time-point of 2006, revealed a non-significant between-country difference in the log_e_ rate of total soft-drink imports (−0.65, 95 % CI: −1.69–0.37; *p* = 0.19) (Table 5). When taken together with the findings in the previous section, these results suggest that increased FDI and soft-drink production in Peru resulted in a reduction in imports into that country following ratification, whereas in Bolivia imports continued to grow steadily. Figure 3 also demonstrates increased imports of soft drinks into Bolivia—a small amount of which were from Peru, rising from <0.1 L per capita in 2006 to 0.058 L in 2012.

**Table 5:** Between-country difference-in-difference estimates for log_e_ rate of change in soft-drink imports per capita before and after ratification (2006) and enforcement (2009) of the US-Peru FTA

Year	Estimate	Unadjusted	Adjusted for GDP	Adjusted for GDP & time trends	N
2006	Diff-in-diff	−0.65 (0.5)	−0.62 (0.51)	−0.66 (0.50)	28
	R^2^	0.07	0.09	0.26	
2009	Diff-in-diff	−0.13 (0.41)	−0.13 (0.42)	−0.13 (0.46)	28
	R^2^	0.13	0.04	0.08	

*Notes:* robust standard-errors in parentheses

### Comparing changes in soft-drink sales volumes and sugar from soft drinks volumes

**Fig. 4:**
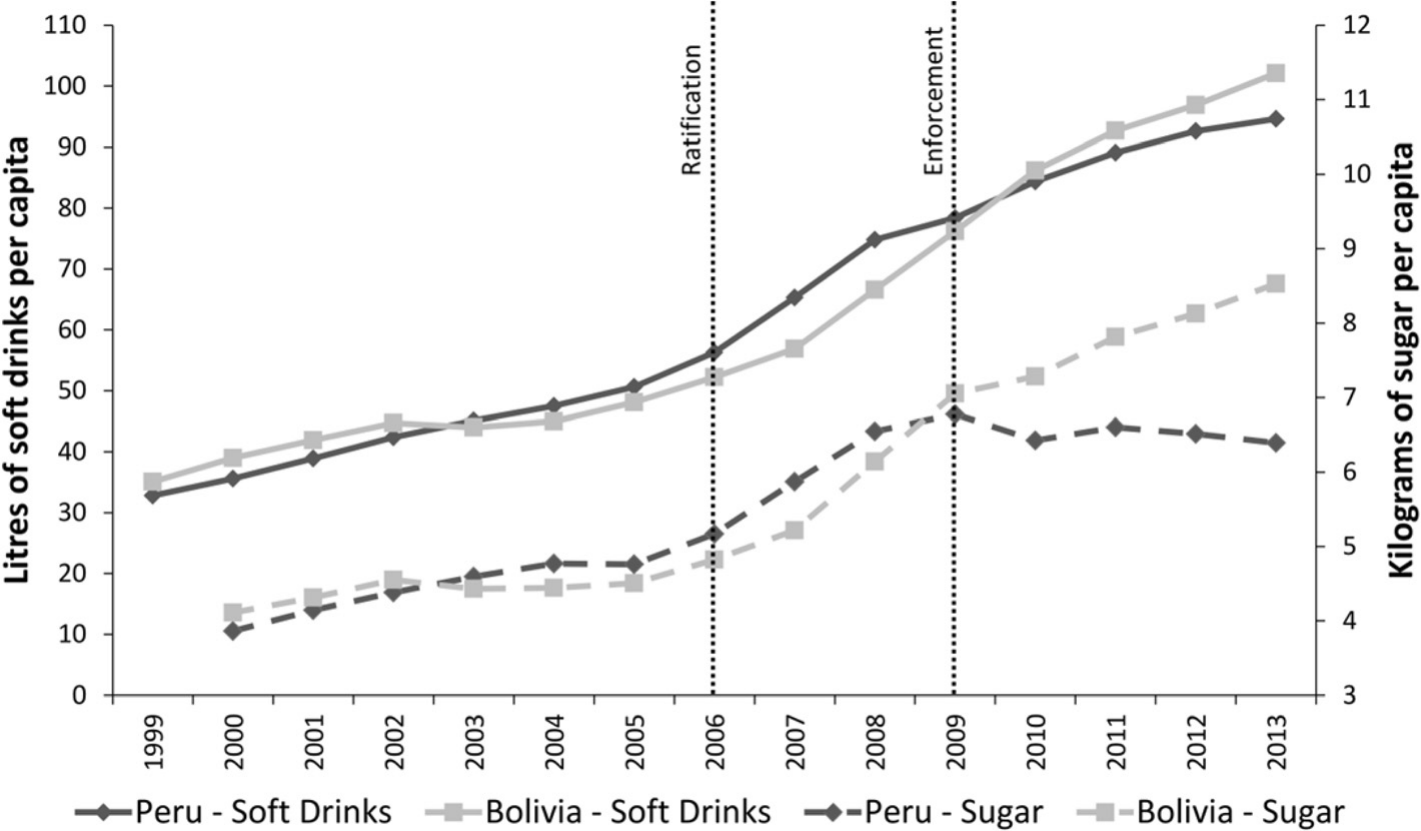
Trends in soft-drink sales volumes per capita and kilograms of sugar from soft-drinks volumes per capita in Peru and Bolivia, before and after ratification (2006) and enforcement (2009) of the US-Peru FTA. *Footnotes:* Data from [19]

The third hypothesis was that changes in FDI-inflows, production and imports would result in a significant change in soft-drink sales volumes in Peru and that this would be synonymous with a change in sugar from soft-drinks volumes. Figure 4 demonstrates changes in the respective countries’ soft-drink sales volumes and changes in sugar from soft-drinks volumes. There was little between-country difference in soft-drink sales volumes per capita. The DID model revealed no significant between-country difference in the log_e_ rate of change in soft-drink sales volumes per capita (Table 6). Although sugar from soft-drinks volume per capita in Peru increased from 4.64Kg (95 % CI: 4.33–4.95) in the pre-ratification period to 6.45Kg (95 % CI: 6.27–6.64) post-ratification, sales volumes stagnated from 2009 onwards. In Bolivia sugar from soft-drinks volume per capita continued to increase from 4.51Kg (95 % CI: 4.37–4.65) to 7.38Kg (95 % CI: 6.50–8.26) respectively. The DID model, with an intervention time-point of 2006, revealed a significant between-country difference in the log_e_ rate of change in sugar from soft-drinks volume per capita (−0.99, 95 % CI: −1.92–0.06, *p* = 0.028) robust to adjustment for GDP and underlying time trends (Table 6). The DID model with an intervention time point of 2009 was also significant (−1.08, 95 % CI: −2.06–0.1, *p* = 0.025) and robust to adjustments for GDP and underlying time trends.

**Table 6:** Between country difference-in-difference estimates for log_e_ rate of change in soft-drink sales volumes per capita and Log sugar from soft-drinks volumes per capita before and after ratification (2006) and enforcement (2009) of the US-Peru FTA

Category	Year	Estimate	Unadjusted	Adjusted for GDP	Adjusted for GDP & time trends	N
Soft drinks	2006	Diff-in-diff	−0.41 (0.36)	−0.41 (0.38)	−0.35 (0.32)	30
		R^2^	0.06	0.06	0.44	
	2009	Diff-in-diff	−0.47 (0.32)	−0.44 (0.33)	−0.45 (0.37)	30
		R^2^	0.19	0.21	0.24	
Sugar from soft-drinks	2006	Diff-in-diff	−0.99* (0.45)	−0.99* (0.50)	−0.98* (0.50)	30
		R^2^	0.19 (*p* = 0.28)	0.19 (*p* = 0.047)	0.29 (*p* = 0.049)	
	2009	Diff-in-diff	−1.08* (0.48)	−1.07* (0.52)	−1.10* (0.51)	30
		R^2^	0.37 (*p* = 0.025)	0.37 (0.039)	0.47 (0.03)	

*Notes:* *p < 0.05; standard-errors in parentheses

### Explaining divergences in sugar from soft-drinks volumes

**Fig. 5:**
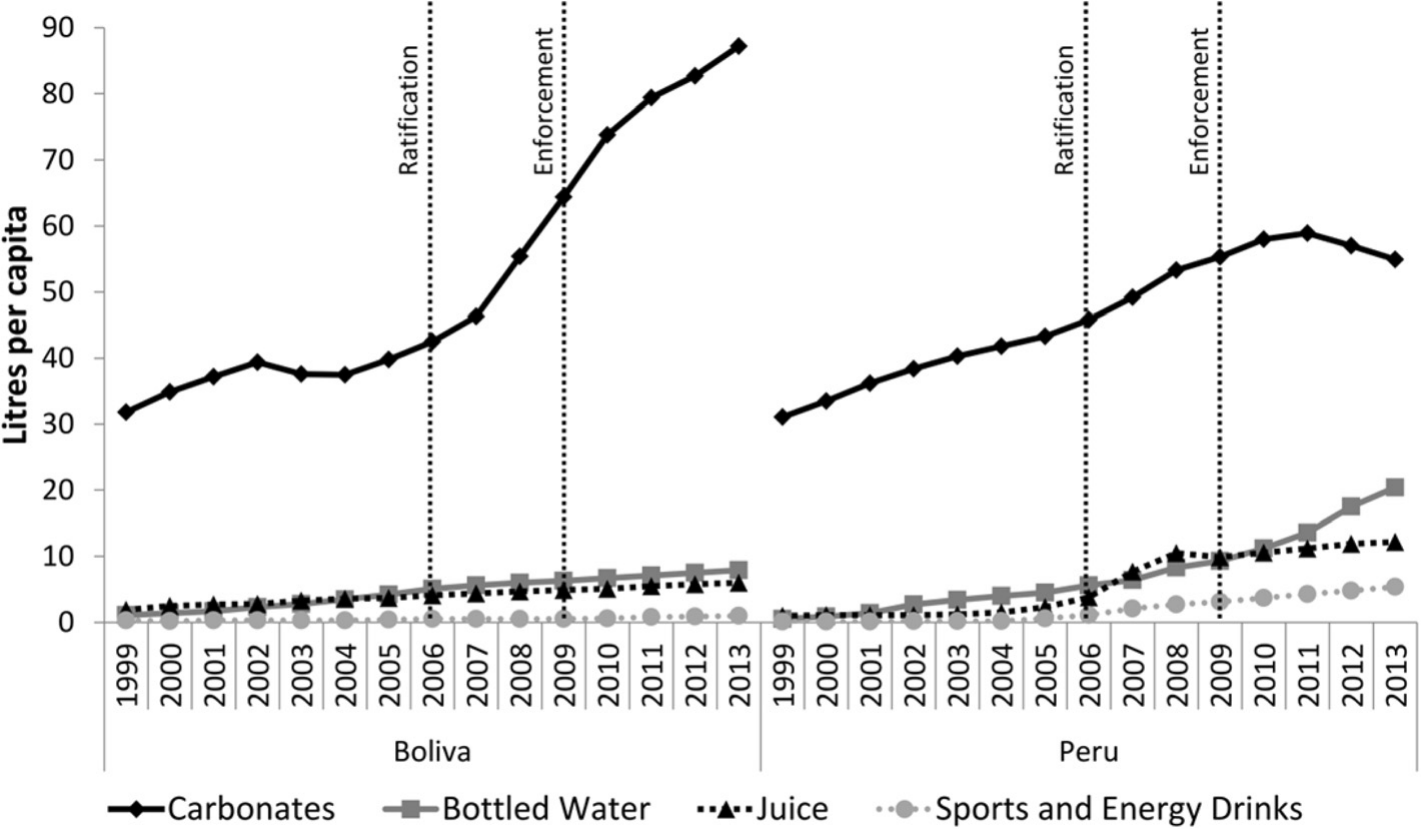
Soft-drink category sales volumes per capita in Peru and Bolivia before and after ratification (2006) and enforcement (2009) of the US-Peru FTA. *Footnotes:* Data from [19]

**Fig. 6:**
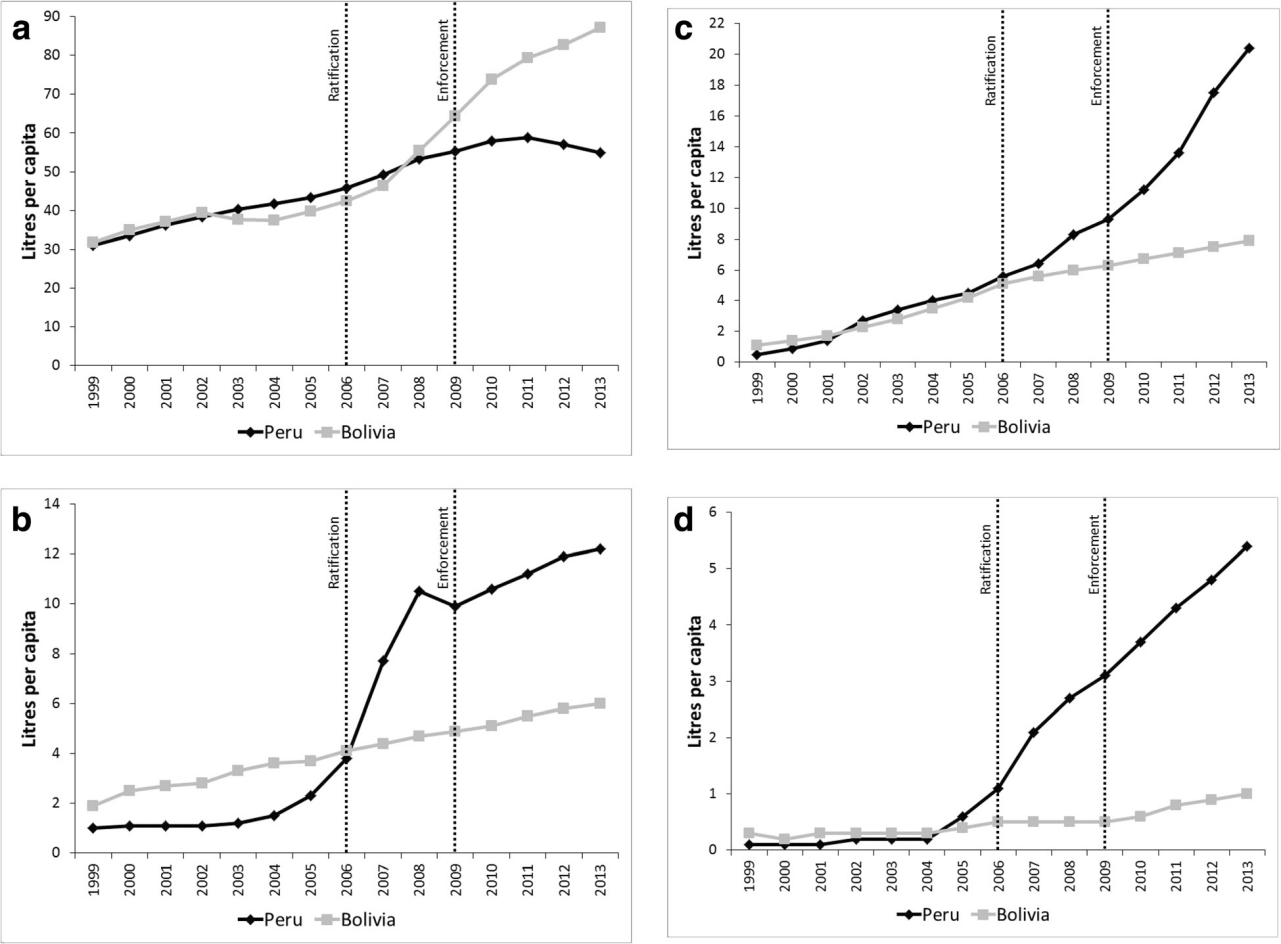
**a**–**d** Soft drink category sales volumes per capita in Peru and Bolivia for the respective categories before and after ratification (2006) and enforcement (2009) of the US-Peru FTA. **a**) carbonates; **b**) juice; **c**) bottled water; **d**) sports & energy drinks. *Footnotes:* Data from [19]

We further hypothesised that the observed divergences in sugar from soft-drinks volumes may reflect changes in the types of soft-drinks being manufactured and sold in Peru. Figure 5 demonstrates changes in sales volumes per capita for the various soft-drink categories. The observed change in the Peruvian market was largely due to increases in bottled water, juice and sports & energy drinks sales volumes, whereas from 2010 onwards carbonates sales volume stagnated. This suggests that stagnated sugar from soft drinks volumes in Peru was largely due to a change in the types of soft drinks produced and sold. The change in the Bolivian soft-drink market was almost exclusively due to an increase in carbonated soft-drinks sales volumes which exceeded per capita volumes in Peru from 2008 onwards. Per capita sales volumes for each category are also given in Fig. 6a–d, demonstrating clear divergences between the two country markets. Between-country DID models were statistically significant with an intervention time-point of 2006 for the log_e_ rate of change in carbonated soft-drinks (−1.44, 95 % CI: −2.52–0.36, *p* = 0.006), robust to adjustments for GDP and underlying time trends. Between-country DID models were statistically significant with an intervention time-point of 2009 for the log_e_ rate of change in bottled water (0.63, 95 % CI: −0.01–1.26, *p* = 0.043), robust to adjustments for GDP and underlying time trends (Table 7).

**Table 7:** Between country difference-in-difference estimates for log_e_ rate of change in soft drink category sales volumes per capita before and after ratification (2006) and enforcement (2009) of the US-Peru FTA

	Year	Estimate	Unadjusted	Adjusted for GDP	Adjusted for GDP & time trends	N
Carbonates	2006	Diff-in-diff	−1.44*** (0.53)	−1.46*** (0.56)	−1.39*** (0.48)	30
		R^2^	0.30 (*p* = 0.006)	0.30 (*p* = 0.009)	0.57 (*p* = 0.004)	
	2009	Diff-in-diff	−1.36*** (0.50)	−1.35*** (0.51)	−1.35** (0.58)	30
		R^2^	0.37 (*p* = 0.006)	0.38 (*p* = 0.009)	0.38 (*p* = 0.019	
Bottled water	2006	Diff-in-diff	0.62* (0.32)	0.59* (0.33)	0.62* (0.32)	30
		R^2^	0.68 (*p* = 0.051)	0.69 (*p* = 0.07)	0.74 (*p* = 0.051)	
	2009	Diff-in-diff	0.63** (0.31)	0.59* (0.34)	0.63** (0.26)	30
		R^2^	0.45 (*p* = 0.043)	0.47 (*p* = 0.085)	0.77 (*p* = 0.016)	
Juice	2006	Diff-in-diff	0.09 (0.81)	0.48 (0.69)	0.53 (0.66)	30
		R^2^	0.04	0.47	0.55	
	2009	Diff-in-diff	−0.47 (0.60)	−0.02 (0.52)	−0.21 (0.55)	30
		R^2^	0.04	0.53	0.55	
Sports & energy drinks	2006	Diff-in-diff	0.12 (0.86)	0.27 (0.73)	0.26 (0.75)	30
		R^2^	0.11	0.34	0.37	
	2009	Diff-in-diff	−0.90 (0.65)	−0.63 (0.58)	−0.67 (0.63)	30
		R^2^	0.13	0.35	0.38	

*Notes:* *p < 0.1; **p < 0.05; ***p < 0.01, standard-errors in parentheses

## Discussion

This analysis revealed two main findings. First, relative to Bolivia, foreign direct investment (FDI) in-flows into Peru increased significantly following ratification and enforcement of the US-Peru FTA. This corresponded with a non-significant decline in soft drinks imports into Peru, with no change observed in Bolivia. The increased FDI-inflows into Peru corresponded with a sustained increase in soft-drink production. This suggests that the FTA may have shaped Peru’s soft-drink market by way of encouraging FDI by US transnational soft-drink corporations and that resulting increased production led to less reliance on imports. This finding supports the view that although cross-border trade of goods remains important, FDI is now a key strategy used by transnational beverage corporations (TBCs) to achieve market share in developing countries. In this case, such FDI likely went into establishing manufacturing plants and other commercial operations of US TBCs [14, 34–36]. These results are consistent with the findings of other analyses demonstrating significant changes in FDI by transnational food and beverage corporations in Mexico following NAFTA, and in Central American countries following CAFTA, both FTAs with the United States [22, 27]. Interestingly we also observed an increase, albeit small, in soft drink imports into Bolivia from Peru following enforcement, suggesting the US-Peru FTA may have had regional effects on soft drink sales.

The second finding supports the general hypothesis that trade agreements can influence the volumes and types of foods and beverages available for purchase and sold, and nutrients consumed. Although the increased total per capita soft-drink sales volume observed in Peru was not in excess of that observed in Bolivia, there was a noticeable change in the types of soft-drinks sold. This was characterised by stagnated growth in carbonated soft drinks, which are predominantly sugar-sweetened and expanded growth in the bottled water, juice and sports & energy drinks categories, with the latter two categories also known to be high in added sugar. In parallel we observed stagnated growth in sugar from soft-drinks volume in Peru, whereas in Bolivia this continued to increase alongside carbonated soft drinks sales volume. This suggests that the increase in FDI in-flows and production was associated with a change in the types of beverages manufactured and sold in Peru. Although the observed increase in bottled water sales in Peru may have had positive effects for public health if consumed as a substitute for sugar-sweetened beverages, this may have been off-set partly by increased sales of the high sugar categories juice and sports & energy drinks. However, it is important to note that the study design makes it difficult to draw inferences and the results may simply reflect changes in the strategic decisions made by soft-drink manufacturers in response to consumer demand for different products rather than changes in FDI in-flows and production resulting from the FTA. Others have also acknowledged the difficulty of separating the effects of trade liberalization from other social and economic influences on food markets and consumer behaviour [22].

There are several other limitations of this analysis that are important to consider. It is not feasible to conduct an experimental intervention to assess the effects of trade and investment policy on food systems and dietary outcomes [25]. Natural experiment designs can produce empirical evidence in situations where an experimental manipulation of the intervention is unfeasible, but when it is important to understand the health impacts of policies and other large-scale phenomena [26, 30]. We used this design in a previous analysis that found a significant increase in sugar-sweetened carbonated soft drink sales volumes in Vietnam following that country’s removal of FDI restrictions subsequent to its accession to the WTO in 2007, in contrast to a control country the Philippines which acceded in 1995 [25]. However, in contrast to Peru, which had stages of unilateral and multi-lateral liberalization prior to the US-Peru FTA, Vietnam was a highly protected market prior to its WTO accession. Thus the US-Peru FTA was not a clear intervention point but additive to prior liberalization events. We justified this by inferring from previous analyses (described in the background section) that an FTA with the US, home to the world’s two largest transnational beverage companies, would likely produce significant changes in Peru’s soft drink market beyond the effects of prior liberalization events.

Another challenge in using the natural experiment design in this analysis was to determine where to introduce the intervention time-point. For example, although the US-Peru FTA was ratified in 2006 and enforced in 2009, changes in soft-drink markets may have occurred earlier when, for example, the Office of the US Trade Representative notified US Congress of the Government’s intention to initiate negotiations with Peru in November 2003, with negotiations beginning in May 2004. Because this may have enhanced US investor confidence in Peru (a so-called ‘market signal’) trade and investment flows may have begun as early as 2004. The staggered timing of the agreement and associated processes (e.g. notification of intent to congress → negotiation → ratification → enforcement) made it difficult to define a single intervention time-point. For these reasons we included two time-points in our models: 2006 as the ratification year and 2009 as the enforcement year.

Future analyses may be strengthened by using a number of falsification tests, through the use of a synthetic control group (e.g. a composite of multiple countries) rather than a single control country, and by controlling for other variables that can shape a given country’s in-ward investment and trade flows. In addition to the variables controlled for in this analysis, these can include, for example, proximity to major markets, economic and political stability, wage-rates, corporate tax structures and other financial (dis)incentives [25, 37]. To the best of our knowledge there were no discernible changes in these factors in either Peru or Bolivia during the selected time-period. To overcome the limitations of the small-N natural experiment design adopted in this analysis, future analyses could adopt medium- to large-N statistical designs that test associations between the ‘depth’ of provisions in trade agreements among all trade partners, with food systems and nutrition measures in all countries for which comparable data is available.

Additional data limitations make clear inferences from this analysis difficult. It was not possible to obtain industry-specific FDI data and the reported trends may therefore reflect FDI in other industries. However, the observed corresponding increase in soft-drink production in Peru suggests that a significant proportion of new FDI-inflows was in the soft-drink industry [38]. Further, we did not capture changes in sales volumes containing non-caloric soft drinks, although this category is likely to be consumed in significant volumes. Nonetheless, the ‘sugar from soft drinks’ variable allowed us to quantify trends in sugar from sugar-sweetened beverages irrespective of trends in non-caloric soft drinks. Finally, per capita sales volume is a proxy measure of consumption and does not capture other sources of soft drinks including those from informal (non-market) sources, nor does it account for wastage (i.e. the proportion of sales not consumed).

## Conclusions

Using the example of Peru and Bolivia, this analysis demonstrates two ways in which FTAs can influence the domestic food environment, thereby affecting the nutritional makeup of the local food supply, with implications for diet-related health. The analysis highlights the important FDI pathway, which is now the primary strategy used by transnational food and beverage corporations to achieve market share in developing country markets and a key mechanism by which FTAs affect food availability and consumption. The analysis also demonstrates that FTAs can influence the diversity and volumes of beverages produced and available for consumption. In the case of Peru this appears to have had both positive and negative implications for nutrition, with stagnated sugar-sweetened carbonated beverages sales and an increase in non-caloric bottled water, but increases in the high-sugar categories juice and sports & energy drinks. However, limitations of the qausi-natural experiment design adopted in this analysis makes inferences difficult and these conclusions should be interpreted with caution.

## Abbreviations

CAFTA, US-Central America Free Trade Agreement; CCC, Coca-Cola Company; CVD, cardiovascular disease; DID, difference-in-difference; FDI, Foreign Direct Investment; FTA, free trade agreement; LMICs, low- and middle-income countries; NAFTA, North American Free Trade Agreement; NCDs, non-communicable diseases; TFBCs, Transnational Food and Beverage Organizations; TPP, Trans-pacific partnership agreement; US, United States; US-Peru FTA, United States – Peru Trade Promotion agreement; WTO, World Trade Organization

## References

[1] Swinburn B (2007). Diet, nutrition and the prevention of excess weight gain and obesity. Public Health Nutr.

[2] World Health Organization (2003). Diet, nutrition and the prevention of chronic diseases. report of a joint WHO/FAO expert consultation.

[3] Basu S (2013). Relationship of soft drink consumption to global overweight, obesity, and diabetes: a cross-national analysis of 75 countries. Am J Public Health.

[4] Malik VS (2010). Sugar-sweetened beverages, obesity, type 2 diabetes mellitus, and cardiovascular disease risk. Circulation.

[5] Hawkes C (2007). Globalization, food and nutrition transitions.

[6] Kennedy G, Nantel G, Shetty P, Food & Agricultural Organization of the United Nations (2004). Globalization of food systems in developing countries: A synthesis of country case studies.

[7] Euromonitor International (2014). Passport global market information database.

[8] Bailey S. Advertising is a key strategy for Coca-Cola’s growth. 2014 [cited 2015 26 March]; Available from: http://marketrealist.com/2014/12/advertising-key-strategy-coca-colas-growth/. Accessed 1 Apr 2015.

[9] Monteiro CA, Cannon G (2012). The impact of transnational “big food” companies on the South: a view from Brazil. PLoS Med.

[10] Stuckler D (2012). Manufacturing epidemics: the role of global producers in increased consumption of unhealthy commodities including processed foods, alcohol, and tobacco. PLoS Med.

[11] Moodie R (2013). Profits and pandemics: prevention of harmful effects of tobacco, alcohol, and ultra-processed food and drink industries. Lancet.

[12] Hawkes C (2005). The role of foreign direct investment in the nutrition transition. Public Health Nutr.

[13] Patel RC (2012). Stuffed and starved: the hidden battle for the world food system.

[14] Shaffer E, Brenner J, Houston T. International trade agreements: a threat to tobacco control policy. Tob Control. 2005;14:(2):19–25. 10.1136/tc.2004.007930 PMC1766197 16046697

[15] Stuckler D (2008). Population causes and consequences of leading chronic diseases: a comparative analysis of prevailing explanations. Milbank Q.

[16] Lang T, Heasman M. Food wars: the global battle for minds, mouths, and markets. London; Sterling: Earthscan; 2004.

[17] Gleeson D, Friel S (2013). Emerging threats to public health from regional trade agreements. Lancet..

[18] Thow AM (2015). Will the next generation of preferential trade and investment agreements undermine prevention of noncommunicable diseases? A prospective policy analysis of the Trans Pacific Partnership Agreement. Health Policy.

[19] Schott J, Kotschwar B, Muir J (2013). Understanding the trans-pacific partnership. Policy Analaysis in International Economics 99.

[20] Friel S (2013). A new generation of trade policy: potential risks to diet-related health from the Trans Pacific Partnership agreement. Glob Health.

[21] Baker P, Kay A, Walls H (2014). Trade and investment liberalization and Asia’s noncommunicable disease epidemic: a synthesis of data and existing literature. Glob Health.

[22] Clark SE (2012). Exporting obesity: US farm and trade policy and the transformation of the Mexican consumer food environment. Int J Occup Env Heal.

[23] Thow AM, Hawkes C (2009). The implications of trade liberalization for diet and health: a case study from Central America. Glob Health.

[24] Schram A, Labonté R, Sanders D (2013). Urbanization and international trade and investment policies as determinants of noncommunicable diseases in Sub-Saharan Africa. Prog Cardiovasc Dis.

[25] Schram A (2015). The role of trade and investment liberalization in the sugar-sweetened carbonated beverages market: a natural experiment contrasting Vietnam and the Philippines. Glob Health.

[26] Craig P (2012). Using natural experiments to evaluate population health interventions: new Medical Research Council guidance. J Epidemiol Commun H.

[27] Hawkes C, Thow AM (2008). Implications of the Central America-Dominican Republic-Free Trade Agreement for the nutrition transition in Central America. Rev Panam Salud Publica.

[28] World Health Organization (2014). Noncommunicable diseases country profiles - Peru.

[29] World Health Organization (2014). Noncommunicable diseases country profiles - Bolivia.

[30] Meyer BD (1995). Natural and quasi-experiments in economics. J Bus Econ Stat.

[31] Friel S (2013). Monitoring the impacts of trade agreements on food environments. Obes Rev.

[32] World Bank. World development indicators. 2014. Available from: http://data.worldbank.org/data-catalog/world-development-indicators. Accessed 14 Nov 2014.

[33] Baker P, Friel S (2014). Processed foods and the nutrition transition: Evidence from Asia. Obes Rev.

[34] Reardon T, Timmer C, Evenson RE, Pingali P, Schultz TP (2005). Transformation of markets for agricultural output in developing countries since 1950: how has thinking changed?. Handbook of agricultural economics: agricultural development: farmers, farm production and farm markets.

[35] Bolling C, Somwaru A (2001). US food companies access foreign markets though direct investment. Food Rev.

[36] Hawkes C (2006). Uneven dietary development: linking the policies and processes of globalization with the nutrition transition, obesity and diet-related chronic diseases. Glob Health.

[37] Blomström M, Kokko A, Mucchielli J-L, Herrmann H, Lipsey R (2003). The economics of foreign direct investment incentives. Foreign direct investment in the real and financial sector of industrial countries.

[38] Ghazalian P, Cardwell R (2009). Liberalisation of trade in primary agricultural commodities: expected effects on food processing FDI. Estey Cent J Int Law Trade Policy.

[39] United Nations Conference on Trade and Development. UNCTADSTAT. 2014; Available from: http://unctad.org/en/Pages/Statistics.aspx. Accessed 14 Nov 2014.

[40] Clark SE (2012). Exporting obesity: US farm and trade policy and the transformation of the Mexican consumer food environment. Int J Occup Environ Health.

[41] United Nations. UN Comtrade Database. 2014. Available from: http://comtrade.un.org/data/. Accessed 14 Nov 2014.

